# Association of physical activity and appetite with visual function related to driving competence in older adults

**DOI:** 10.1186/s12877-017-0484-6

**Published:** 2017-04-26

**Authors:** Takafumi Ando, Hiroyuki Sakai, Yuji Uchiyama

**Affiliations:** Human Science Research Domain, Toyota Central R&D Labs., Inc, 41-1 Yokomichi, Nagakute, Aichi, 480-1192 Japan

**Keywords:** Lifestyle habit, Physical activity, Accelerometry, Appetite, Driving safety, Useful field of view, Aging, Cognitive decline

## Abstract

**Background:**

Older people are at greater risk of traffic accidents, partially because of age-related declines in visual function, including reduced useful field of view (UFOV). However, lifestyle factors which cause age-related decline in UFOV remain poorly understood. We conducted a study to investigate whether physical activity and appetite status were related to UFOV test performance in healthy older adults.

**Methods:**

Thirty community-dwelling older people (age 68.6 ± 3.1 years, 15 females) were enrolled in this study. Each participant completed the Council on Nutrition appetite questionnaire (CNAQ) and a UFOV test. They then wore a tri-axial accelerometer (Active style Pro HJA-350IT) for 3–6 consecutive days to objectively measure their PA in free-living conditions.

**Results:**

Longer time spent in vigorous physical activity was significantly associated with better UFOV test performance when adjusted for age and accelerometer wear time (non-locomotive: *r* = −0.435, locomotive: *r* = −0.449; *n* = 25). In addition, male, but not female, participants with a higher CNAQ score had significantly better UFOV test performance in both an unadjusted model (*r* = −0.560; *n* = 15) and a model adjusted for age (*r* = −0.635; *n* = 15).

**Conclusions:**

The results suggest that appetite status among males and time spent in high intensity PA are associated with visual function related to driving competence in older adults.

## Background

Elderly people are more at risk of traffic accidents. In Japan, fatal accident rate increases with age in elderly drivers [1] and pedestrians [2]. When navigating through a traffic environment, a road user is required to interpret traffic scenes and choose appropriate actions without premeditation. However, it is well known that various functions, including sensory, perceptual, cognitive, and motor functions, gradually decline as part of the normal aging process. Many researchers have therefore focused on the impact of such age-related functional decline on the risk of traffic accidents [3].

The useful field of view (UFOV) assessment is widely accepted as a measure of visual function that predicts driving competence in older adults [4]. The UFOV test was originally designed to capture the age-related decline in the visual area over which necessary information can be obtained in a brief glance [5]. Subsequent retrospective [6], prospective [7], and interventional [8] studies demonstrated that visual function assessed using the UFOV test was associated with at-fault crash risk in older drivers. Although there was considerable variation in the stimulus displays and scoring methods of the UFOV assessments in the early studies, a standardized version (UFOV®) is now commercially available. This standardized UFOV test includes three subtests: subtest 1 measures processing speed (discriminating stimuli presented in central vision), subtest 2 measures divided attention (subtest 1 with concurrent detection of a peripheral stimulus), and subtest 3 measures selective attention (subtest 2 with additional distractions). Recent studies [9, 10] have shown that the divided attention subtest may be an adequate measure of visual function related to driving competence in older adults. The aspects of visual functioning assessed by the divided attention component of the UFOV test include not only divided attention but also sensory, perceptual, and cognitive abilities.

The individual variation in visual function evaluated by the UFOV test is considerable and potentially critical for safe driving by older adults. However, the factors that cause this variation are not well understood. Recently, the beneficial effects of active lifestyles on functional health in the elderly have become apparent [11, 12]. The results of three studies that investigated the relationship between physical activity (PA) and UFOV in older adults were inconsistent. Two studies found a significant association between PA and UFOV test performance [13, 14], but the other reported no association [15]. These observational studies used questionnaires to evaluate PA, which may not be a reliable or accurate method to assess PA [16, 17]. In addition, these studies did not precisely evaluate the mode, intensity, and duration of PA.

Poor appetite in older adults could be another factor contributing to age-related cognitive decline. Age-related decrease in appetite, called “anorexia of aging”, is considered a potential cause of malnutrition in older adults [18–20]. With respect to malnutrition and cognitive decline, several studies have reported that healthy dietary patterns in older adults were associated with less cognitive decline [21]. These previous studies imply that older adults with lower appetites have lower cognitive performance than those with higher appetites. In fact, Ohrmann et al. indicated that younger anorexia patients showed lower performance on a divided attention task than did healthy controls [22]. However, there is no direct evidence of the association between appetite and UFOV test performance in older adults.

We conducted a pilot study to investigate whether PA, assessed using a tri-axial accelerometer, was associated with UFOV test performance in healthy older individuals. We investigated whether appetite status was related to UFOV test performance in older adults, using the Council on Nutrition appetite questionnaire (CNAQ) [23].

## Methods

### Participants

Thirty participants (15 females and 15 males, mean age 68.6 ± 3.1 years) enrolled in this study. Healthy older people aged between 65 and 74 years were recruited through local help-wanted magazines distributed in Nagoya, Japan, and the surrounding suburbs. All participants reported being free of eye diseases (glaucoma, cataract, and macular degeneration), severe lower back pain, gait difficulty, history of psychiatric disorders, diabetes mellitus, and photosensitive epilepsy. According to results of the mini-mental state examination, no participants showed severe general cognitive decline (mean score: 28.3 ± 1.7, range: 24–30). In addition, no participants had regular water-based exercise, such as swimming.

Each participant provided written informed consent before enrollment. The study protocol was approved by the institutional ethics committee of Toyota Central Research and Development Laboratories, Inc. This study was conducted between late September 2014 and early November 2014.

### UFOV test

Participants completed a computer-controlled UFOV assessment seated in front of an LCD monitor (FG2421, EIZO Corporation, Ishikawa, Japan) at a visual distance of 60 cm. The spatial and temporal resolutions of the monitor were 1280 × 1024 pixels and 100 Hz, respectively. The UFOV assessment was controlled by Psychtoolbox software with GNU Octave 3.2.4, using the Linux operating system (Ubuntu 12.04 LTS).

**Fig. 1:**
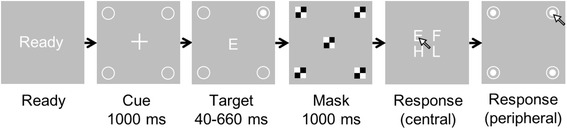
Stimuli in the useful field of view test. The target stimulus was a letter (E, F, H, or L) and a filled circle in one corner while the participant fixated on the center of the screen. After the stimuli were masked, the participant indicated the letter and circle position. The trials in which participants correctly identified both the letter and the position were considered to be correct

We adopted the divided attention subtest of the UFOV assessment (Fig. 1), which had been proposed in earlier research [24]. The appearance of this UFOV test was different from the commercial version. We confirmed that performance on this test was a good predictor of driving competence in older adults in our previous study [10].

At the beginning of each trial, the word “Ready” appeared at the center of the monitor. When the participant pressed a mouse button, a fixation crosshair appeared, with four open circles at the corners of the screen (eccentricity =12.6°). After 1000 ms, the fixation cross was replaced with a letter (E, F, H, or L) as a central target, and a solid circle was simultaneously presented inside one of the open circles as a peripheral target. The target duration was randomly selected from 40, 60, 100, 180, 340, and 660 ms. The central letter and all four circles were then masked with a checkerboard pattern for 1000 ms, after which the participant was instructed to use the mouse to indicate the central target letter and the position of the peripheral target. One session consisted of 48 trials. The participants completed six sessions, with an inter-session interval of <10 min. Before the sessions started, the participants practiced the task with two longer target durations (1000 and 500 ms) to acclimate to the task.

The trials in which participants correctly identified both the central and peripheral targets were considered to be correct. For each target duration, the percentage of correct trials was calculated. Then a logistic model was created for the percentage of correct trials as a function of target duration using the glmfit command in Matlab 2014a (MathWorks, Inc., MA, USA) with a binomial distribution. UFOV test performance was defined as the shortest target duration with a percentage correct ≥53% (= (1 + 1/16)/2) on the fitted logistic function. Shorter duration represented better UFOV performance.

### Assessment of appetite status and physical activity

Before the UFOV test, each participant completed the 8-item CNAQ [23], which was translated into Japanese for the present study. The CNAQ included questions about subjective regular feeling of appetite, taste, sickness, mood, and meal frequency. Responses were scored with a 5-point Likert-type scale (e.g., My appetite is: 1 = very poor, 2 = poor, 3 = average, 4 = good, and 5 = very good). After the UFOV test, participants were instructed to wear a tri-axial accelerometer (Active style Pro HJA-350IT; Omron Healthcare, Kyoto, Japan) for 3–6 consecutive days to evaluate their habitual PA in free-living conditions. They wore the accelerometer on their right front waist during waking hours except during water-based activities. The accelerometer was developed especially for the separate evaluation of non-locomotive and locomotive PA, with a wide range and high resolution of intensity [25]. The accelerometer recorded PA in metabolic equivalents (METs) for each 10 s time window (10 s epochs).

The recorded PA data were first divided into wear and non-wear periods, with the latter defined as periods of ≥60 consecutive minutes of zero METs. The remaining periods with zero METs were assigned a value of 0.9 METs as a basal metabolic rate, in accordance with our previous study [26]. Participants with at least three valid days, with ≥600 min of wear time per day, were included in the following analysis. The PA data were summarized as the accumulated time spent in each of the following categories: sedentary behavior (≤1.5 METs), non-locomotive and locomotive light PA (1.6–2.9 METs), non-locomotive and locomotive moderate PA (3.0–5.9 METs), and non-locomotive and locomotive vigorous PA (≥6.0 METs). Total non-locomotive time was the sum of light, moderate, and vigorous non-locomotive PA. Total locomotive time was similarly calculated. The breaks in sedentary behavior was the number of interruptions in sedentary behavior during which the intensity of PA increased to >1.5 METs [27]. Finally, the PA level (PAL), the ratio of daily total energy expenditure to basal metabolic rate in the supine position, was calculated as

$$ \mathrm{PAL}=\frac{\int_{T_w} I(t) dt+0.9{\int}_{T_N} dt}{0.9{\int}_{T_W+{T}_N} dt} \cdot {k}_{DIT} $$

where *T*_*W*_ and *T*_*N*_ are the wear and non-wear times, respectively; *I(t)* represents the intensity of PA (in METs) as a function of time; and *k*_*DIT*_ is a constant (1/0.9) that takes into account diet-induced thermogenesis. The daily mean of each PA parameter was calculated for each participant (see Table 1).

**Table 1:** Time spent in physical activity and the correlation with useful field of view

	Mean ± SD	Correlation coefficient	*p* value
Wear time (min)	980.2 ± 159.5	0.207	> 0.1
Physical activity level	1.69 ± 0.18	−0.112	> 0.1
Sedentary behavior (SB)			
Total SB (min)	625.8 ± 133.7	0.330	> 0.1
Light physical activity (LPA)			
Non-locomotive LPA (min)	206.1 ± 59.5	0.018	> 0.1
Locomotive LPA (min)	57.9 ± 26.2	−0.237	> 0.1
Total LPA (min)	264.0 ± 71.2	−0.072	> 0.1
Moderate physical activity (MPA)			
Non-locomotive MPA (min)	48.0 ± 23.2	−0.289	> 0.1
Locomotive MPA (min)	41.2 ± 30.0	0.037	> 0.1
Total MPA (min)	89.2 ± 41.1	−0.135	> 0.1
Vigorous physical activity (VPA)			
Non-locomotive VPA (min)	0.64 ± 0.36	−0.258	> 0.1
Locomotive VPA (min)	0.49 ± 0.93		
log (locomotive VPA + 0.01)		−0.308	> 0.1
Total VPA (min)	1.13 ± 1.07	−0.367	0.071
Total non-locomotive time (min)	254.8 ± 74.8	−0.077	> 0.1
Total locomotive time (min)	99.6 ± 52.0	−0.104	> 0.1
Breaks in sedentary behavior	382.1 ± 84.9	0.075	> 0.1
Step			
Step counts (number)	7638 ± 4420	0.050	> 0.1
Step rate (number/min)	75.3 ± 12.3	0.334	> 0.1

*Abbreviations*: *SD* standard deviation, *log* log base 10

### Statistical analysis

First, we checked the assumption of normality for the distribution of all variables in the present study using the Kolmogorov–Smirnov test with a significance level of 10%. The distributions of UFOV test performance, years of education, and locomotive vigorous PA significantly deviated from the normal distribution. These variables were normalized with a log_10_ transformation prior to further statistical analysis. We added 0.01 to the locomotive vigorous PA values for all participants to avoid divergence for seven participants with zero values. Second, we performed an analysis of covariance with sex as the main effect to establish whether there were sex interactions between PA variables or CNAQ score and UFOV test performance. If there was an interaction, then the association between PA variables or CNAQ score and UFOV test performance would have been stratified by sex.

To determine whether UFOV test performance was associated with PA variables or CNAQ score, Pearson’s correlation coefficients were calculated. Subsequently, a Pearson partial correlation analysis that adjusted for confounding factors was also performed for each variable. Confounders were selected by the stepwise Akaike information criterion method. The potential confounders were CNAQ, sex, age, years of education, and wear time of accelerometer for each PA variable and the PA variables, age, and years of education for CNAQ.

In addition, we performed a Pearson correlation analysis to examine the effects of aging on PA variables, CNAQ score, and UFOV. We also examined sex differences in PA variables, CNAQ score, and UFOV using two-sample *t*-tests.

Correlations and comparisons were considered statistically significant when *p* < 0.05. All data are reported as mean ± *SD*.

## Results

### Participant characteristics, sex differences, and aging effects

All participants completed the UFOV test and the mean test performance was 353.6 ± 246.1 ms. The participants completely answered the CNAQ, with a mean score of 30.1 ± 2.6. Five participants did not meet the inclusion criterion for the PA assessment (i.e., at least three valid wear days). A total of 25 participants (10 females and 15 males) were included in the subsequent analysis. Table 1 shows the mean values for the PA variables. The mean number of wear days and wear time were 4.7 ± 1.2 days and 980.2 ± 159.5 min/day (16.3 ± 2.7 h/day), respectively.

Neither UFOV nor CNAQ score differed between males and females (both *p* > 0.05). In contrast, wear time, sedentary behavior, breaks in sedentary behavior, non-locomotive light PA, and total non-locomotive time were significantly greater in females than in males (All *p* < 0.05). No other PA variables showed a significant sex difference.

We also analyzed aging effects. PAL, total light PA, breaks in sedentary behavior, total non-locomotive time, and log UFOV test performance declined with increasing age (all *p* < 0.05). Locomotive and non-locomotive light PA, total moderate PA, breaks in sedentary behavior, and total locomotive time tended to decrease with increasing age (all *p* < 0.1). No significant aging effects were found for the other PA variables or CNAQ score (all *p* > 0.1).

### Physical activity and UFOV test performance

**Fig. 2:**
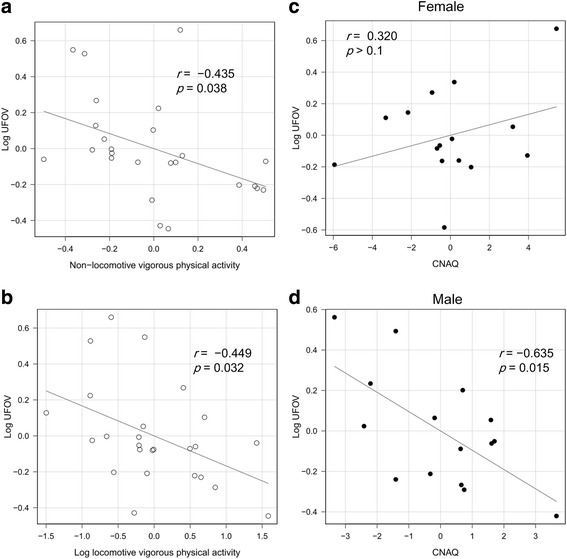
Standard residual plots of lifestyle parameters vs. useful field of view (UFOV) test performance. **a** non-locomotive vigorous physical activity vs. log UFOV test performance, adjusted for age and wear time; **b** log locomotive vigorous physical activity vs. log UFOV test performance, adjusted for age and wear time; **c** CNAQ vs. log UFOV test performance adjusted for age (female participants); **d** CNAQ vs. log UFOV test performance adjusted for age (male participants). CNAQ, Council on Nutrition appetite questionnaire, used to assess appetite status

The analysis of covariance revealed no significant interaction of sex for the associations between any of the PA variables and UFOV test performance. There were no significant simple correlations between any PA variable and UFOV test performance (Table 1). However, the stepwise AIC method found that age and wear time were confounders of the correlation of UFOV test performance with locomotive and non-locomotive vigorous PA. After adjustment for age and wear time, longer non-locomotive and locomotive vigorous PA were significantly associated with better UFOV test performance (*r* = −0.435, Fig. 2a, and *r* = −0.449, Fig. 2b, respectively).

### Appetite status and UFOV test performance

There was a significant interaction of sex for the association between CNAQ score and UFOV (*p* < 0.01) so the correlation analysis for CNAQ score and UFOV was stratified by sex. Among males, a higher CNAQ score was significantly correlated with better UFOV test performance (*r* = −0.560, *p* = 0.03). Based on the stepwise AIC method, age was a confounder. After adjustment for age, the correlation between CNAQ score and UFOV test performance became slightly stronger (*r* = −0.635, *p* = 0.015; Fig. 2d). Among females, there was no significant correlation between CNAQ score and UFOV (*r* = 0.415, *p* > 0.1). Based on the stepwise AIC method, no variables were confounders of this correlation. Figure 2c shows the age-adjusted correlation between CNAQ score and UFOV test performance for females, as was done for males (*r* = 0.320, *p* > 0.1).

## Discussion

To the best of our knowledge, the present study was the first to use a tri-axial accelerometer to investigate the association between PA and UFOV test performance in older adults. Our results showed that vigorous PA was moderately correlated with UFOV test performance in older adults, after adjustment for age and wear time. However, PAL was not correlated with UFOV test performance. This was also the first study to describe the association between appetite status and UFOV test performance. We found that better appetite status was associated with better UFOV test performance in males but not in females.

Our results suggest that PA is associated with visual function related to driving competence in older adults. More specifically, accelerometer data revealed that vigorous intensity PA (≥6 METs) was positively associated with UFOV test performance. The PA other variables (PAL and other PA intensities) were not associated with UFOV. Recently, numerous PA researchers have advocated for increasing PAL and reducing sedentary time for health [28]. However, our study did not reveal a clear association of UFOV with PAL or sedentary behavior. This result was consistent with previous findings, using self-reported PA, that regular moderate to vigorous exercise but not total amount of PA was associated with UFOV test performance [14]. Several recent studies focused on the intensity of exercise or PA in older adults. Some of these studies found that increasing the proportion of vigorous PA reduces the risk of decline in physical functioning [29], coronary events [30], mortality from dementia [31], and all-cause mortality [32]. Recent studies of older adults also revealed a beneficial effect of high intensity intermittent training on muscle protein synthesis [33] and cardiac function in people with coronary heart disease [34] compared with traditional moderate intensity continuous aerobic training. High intensity PA may be needed to maintain visual function in older adults.

However, previous intervention studies found that exercise did not substantially improve UFOV test performance in older adults [35–37], which casts doubt on a causal effect of PA on UFOV test performance. This evidence has two possible interpretations. The first is that acute PA increases in later life cannot improve UFOV due to factors such as poor neural plasticity in older adults and that regular exercise throughout middle age is needed to prevent the decline in UFOV. The present study provided only a cross-sectional view of the association between PA and UFOV test performance. An active individual in the present study may have maintained PA and fitness levels throughout his or her life. In other words, adopting a fairly active lifestyle may be needed to maintain visual function before the start of its decline. The second interpretation relates to the differences in PA intensity in these studies. The present study revealed that PA at a level > 6 METs was associated with better UFOV test performance. In contrast, the previous intervention studies only reported relative exercise intensity, if any (for example, 60–75% of the maximum heart rate). In general, it is hard to impose a level of PA >6 METs as an intervention for older adults because such high intensity is close to maximum capacity for a large proportion of older adults, with the exception of highly trained athletes [38]. In addition, intensive exercise is difficult to implement as an intervention task because it may result in injury, exacerbation of cardiovascular diseases, or locomotor disability. It could therefore be considered that the exercise intensity in the previous interventions was insufficient for the improvement of UFOV test performance. An intervention study that involves high intensity exercise or high intensity intermittent training in older adults is required to clarify the association between vigorous PA and UFOV test performance.

The results of the present study also suggested that appetite status was associated with visual function related to driving competence at least in older males. Recently, health concerns regarding impaired appetite regulation in older adults have become a research focus. Loss of appetite leads to a risk of reduced diet quantity and quality, which in turn may lead to conditions, such as sarcopenia, frailty, and dementia [20]. Interestingly, these appetite-related diseases occur in conjunction with cognitive impairment [39]. Similarly, there is much evidence of the beneficial effects of proper nutrition in the prevention of cognitive impairment [21]. Overall, these findings suggest an association between cognitive decline, malnutrition and appetite. It may be that visual function decline is part of an age-related functional decline due to malnutrition resulting from loss of appetite.

Questionnaire-based assessments of appetite do not necessarily represent malnutrition although a previous study found that CNAQ score might have predicted subsequent weight loss [23]. Dietary record with food weighing or blood biochemistry would be required to more directly study the association between nutritional status and UFOV performance. This is an important challenge for future research to verify the present findings.

The sex interaction in the association between appetite and UFOV is a fascinating result of this study. This result suggests that there is a different mechanism for age-related decline in visual function for each sex. However, the mechanisms for this sex interaction remain an open question, and further investigation that involves the collection of more detailed data, such as dietary records, will be needed to investigate this issue.

A possible mechanism for the association of high-intensity PA and appetite with visual function related to driving competence may be brain plasticity modification. Colcombe et al. [40] indicated that aerobic exercise increased the regional gray matter volume in the brain, including in the right inferior frontal gyrus. A more recent study showed that changes in cardiorespiratory fitness due to a 12-week exercise intervention were positively correlated with cortical thickness changes in the right prefrontal regions [41]. On the other hand, a meta-analysis of patients with anorexia nervosa found reductions in regional gray matter volume in a cluster extending to the right prefrontal region [42]. According to an intervention study with UFOV training done by Scalf et al. [43], the right prefrontal region is associated with increased UFOV performance.

The present study had several limitations. Causality cannot be determined because of the cross-sectional design. The possibility that poor visual function causes poor appetite status or shorter times of vigorous PA cannot be excluded. A longitudinal cohort or intervention study would provide better understanding of the relationship between PA or CNAQ and age-related declines in visual function. In addition, the PA assessment period was short, and the sample size was relatively small. Nevertheless, sampling bias was minimized because the mean PAL in this study was similar to that in a previous Japanese study with more reliable methods (PAL =1.66 ± 0.24) [44].

## Conclusions

Our results suggest that high intensity PA is associated with UFOV performance in older adults. In addition, our data indicate that poor appetite status is associated with UFOV test performance in older males.

Maintaining safe and independent mobility in older adults is important for their quality of life and is also a major factor in self-reliance [45]. Recent social trend run to encouragement of voluntary return of driver’s license; however, driving cessation by older drivers may not be a panacea to solve this issue [46]. Thus, studies aimed at extending life as a driver are required. To this end, intervention studies to demonstrate beneficial effects of daily active lifestyle on elderly driving competence are needed in future work.

## Abbreviations

UFOV: Useful field of view
PA: Physical activity
CNAQ: Council on Nutrition appetite questionnaire
METs: Metabolic equivalents
PAL: Physical activity level
AIC: Akaike information criterion
